# Is a Switch From Insulin Therapy to Liraglutide Possible in Japanese Type 2 Diabetes Mellitus Patients?

**DOI:** 10.14740/jocmr1719w

**Published:** 2014-02-06

**Authors:** Takehiro Kawata, Akira Kanamori, Akira Kubota, Hajime Maeda, Hikaru Amamiya, Masahiko Takai, Hideaki Kaneshige, Fuyuki Minagawa, Kotaro Iemitsu, Mizuki Kaneshiro, Masashi Ishikawa, Hiroshi Takeda, Tetsurou Takuma, Atsuko Mokubo, Hideo Machimura, Mitsuo Obana, Masaaki Miyakawa, Yoshikazu Naka, Daisuke Suzuki, Yasuo Terauchi, Masao Toyoda, Yasushi Tanaka, Ikuro Matsuba

**Affiliations:** a The Study Group of the Diabetes Committee, Kanagawa Physicians Association, 3F Kanagawa-ken Sogo Iryo Kaikan, 3-1 Fujimi-cho, Naka-ward, Yokohama-city, Kanagawa 231-0037, Japan; b Department of Endocrinology and Diabetes, Yokohama City University Medical Center, 4-57 Urafune-cho, Minami-ward, Yokohama-city, Kanagawa 232-0024, Japan; c Division of Nephrology and Metabolism, Department of Internal Medicine, Tokai University School of Medicine, 143 Shimkasuya, Isehara, Kanagawa 259-11, Japan; d Department of Internal Medicine, Division of Metabolism and Endocrinology, St. Marianna University School of Medicine, 2-16-1 Sugao, Miyamae, Kawasaki, Kanagawa 216-8511, Japan

**Keywords:** Type 2 diabetes mellitus, HbA1c, GLP-1 receptor agonist, Liraglutide, Hypoglycemia

## Abstract

**Background:**

To evaluate the efficacy of switching from insulin to the GLP-1 receptor agonist liraglutide in type 2 diabetes mellitus patients.

**Methods:**

The subjects were 231 outpatients with type 2 diabetes mellitus being treated with liraglutide for the first time. For 161 patients, liraglutide was continued for 24 weeks (continuation group), and for 70 patients, liraglutide was discontinued before 24 weeks (discontinuation group). Fasting and postprandial blood glucose levels, HbA1c, body weight, and insulin dose were evaluated before the switch to liraglutide (baseline) and at 12 and 24 weeks of administration. Trends in HbA1c and weight were compared at 12 and 24 weeks of administration. Multiple regression analyses were conducted to identify clinical factors predicting a successful switch to liraglutide.

**Results:**

Multiple regression analysis with ΔHbA1c as the dependent variable in the continuation group indicated that HbA1c at 12 weeks of administration decreased with higher baseline HbA1c and increased with higher baseline daily insulin doses. Multiple regression analysis with Δweight as the dependent variable indicated that Δweight at 24 weeks of liraglutide administration was higher with higher baseline daily insulin doses and longer duration of diabetes. Based on the area under the receiver operating characteristic curve, cut-off values of 19 units for daily insulin dose and nine years for duration of diabetes were identified.

**Conclusions:**

Switching from insulin to liraglutide therapy is possible for carefully selected patients. Daily insulin dosage and duration of insulin therapy appear to be clinically useful indicators for the efficacy of liraglutide therapy.

## Introduction

The appearance of incretin-related drugs as antidiabetic agents has transformed the treatment of type 2 diabetes mellitus (DM). With these agents, the risk of hypoglycemia is low, and weight gain does not occur, making them promising as drugs that will contribute to pre- and postprandial blood glucose control. GLP-1 receptor agonists, in addition to acting to improve blood glucose, control appetite [1], promote regeneration and proliferation of pancreatic β cells, and have extrapancreatic actions such as inhibiting apoptosis [2, 3].

The GLP-1 receptor agonist liraglutide (lira) has been shown in clinical studies outside Japan to significantly decrease HbA1c compared with glimepiride, inhibit weight gain, decrease the risk of hypoglycemia [4] and, in combination with oral hypoglycemic agents, to powerfully improve blood glucose compared with sustained-release insulin [5]. A systolic blood pressure (SBP) lowering effect has also been seen [6, 7]. However, lira is not effective in all type 2 DM patients, and subjects need to be selected carefully.

Unexpectedly, diabetic ketoacidosis and hyperglycemia occurring as a result of switching from insulin therapy to lira were reported at the time of lira’s introduction to Japan. Overall, of the patients given this drug, diabetic ketoacidosis occurred in 4 (2 of whom died), and hyperglycemia occurred in 16 patients. Of these 20 patients, 17 developed the event after switching to lira from insulin therapy. Given this background, it became evident that switching from insulin therapy to lira is appropriate only in selected patients.

However, reports on switching from insulin to lira are currently limited. Therefore, in the present study, the clinical profiles of Japanese type 2 DM patients who were switched from insulin to lira by a diabetes specialist as part of routine outpatient care were retrospectively collected and analyzed. Trends in factors including blood glucose control, body weight, and adverse drug reactions were observed for 6 months after switching from insulin therapy, and the clinical background factors that were associated with a successful switch from insulin therapy to lira were investigated.

## Materials and Methods

### Ethics statement

The protocol of this study was approved by the Ethics Review Board of the Kanagawa Physicians Association. All subjects gave their informed consent prior to their inclusion in the study.

### Subjects

The subjects were 231 patients (137 men, 94 women) switched from insulin to lira who were ≥ 20 years old and whose HbA1c (National Glycohemoglobin Standardization Program (NGSP) values and International Federation of Clinical Chemistry (IFCC) units) could be followed for 6 months, from among the type 2 DM patients receiving outpatient treatment at 18 medical institutions that specialize mainly in diabetes and have on staff a member of the Diabetes Control Committee of the Kanagawa Physicians Association. The subjects were outpatients with type 2 DM treated for the first time with lira between June 2010 and October 2011. Subjects were excluded from the study if they had a history of type 1 DM, or if they were treated for less than 24 weeks or with oral hypoglycemic agents (OHAs) when lira was started, based on the indication accepted by Japanese health insurance. The lira dose was increased from 0.3 to 0.9 mg/day in weekly increments of 0.3 mg. The maximum permitted dosage of lira is 0.9 mg/day in Japan.

### Methods

The 231 patients enrolled were divided into two groups: 161 patients (93 men, 68 women, mean age 60 ± 11 years) in whom lira was continued until 24 weeks after it was started (continuation group); and 70 patients (44 men, 26 women, 60 ± 11 years) in whom lira was discontinued before 24 weeks (discontinuation group). Evaluations were made of fasting blood glucose (FBG), postprandial blood glucose (PBG), and HbA1c levels, body weight (BW), and insulin dose before the switch to lira at baseline (0 weeks) and at 12 weeks and at 24 weeks of lira administration. The trends in HbA1c and BW were compared at 12 and 24 weeks of lira administration, together with an evaluation of adverse events such as hypoglycemia. The OHAs used at the time of the switch are shown in Table 1.

**Table 1:** Characteristics of the Enrolled Population and Subject Disposition

	Liraglutide continuation group	Liraglutide discontinuation group	P value
	n = 161	n = 70	
Sex: male/female (%)	58/42	62/38	P > 0.05
Age (years)	60 ± 11	60 ± 11	P > 0.05
Duration of diabetes (years)	11 ± 8	14 ± 9	P < 0.01
SBP (mmHg)	128 ± 17	124 ± 14	P > 0.05
DBP (mmHg)	74 ± 12	72 ± 11	P > 0.05
Cr (mg/dL)	0.82 ± 0.31	0.85 ± 0.31	P > 0.05
TC (mg/dL)	183 ± 31	191 ± 33	P > 0.05
LDL (mg/dL)	107 ± 29	104 ± 29	P > 0.05
HDL (mg/dL)	54 ± 13	55 ± 19	P > 0.05
TG (mg/dL)	147 ± 82	175 ± 184	P > 0.05
BMI (kg/m^2^)	26.1 ± 4.9	26.8 ± 5.0	P > 0.05
ΔBW (0 - 12 weeks)	-1.6 ± 2.8	-0.8 ± 2.9	P < 0.001
HbA1c (%, mmol/mol)	7.3 ± 1.2, 56 ± 13.1	8.0 ± 1.4, 64 ± 15.3	P < 0.01
ΔHbA1c (0 - 12 weeks) (%, mmol/mol)	-0.4 ± 0.3, -4.4 ± 3.3	0.6 ± 0.9, 6.6 ± 9.8	P < 0.001
Insulin therapy (BOT/Premix/Basal bolus) (%)	27/13/60	20/6/74	P > 0.05
Total insulin dose (U/day)	18 ± 15	29 ± 17	P < 0.001
Prestudy OAD treatment (n)			
SU/BG/TZD/αGI/glinide/none	28/24/15/27/14/96	11/13/6/7/3/46	P > 0.05

Data are means ± SD or n (%), N (number) unless otherwise noted. SBP: systolic blood pressure; DBP: diastolic blood pressure; Cr: creatinine; TC: total cholesterol; LDL: low-density lipoprotein; HDL: high-density lipoprotein; TG: triglycerides; BMI: body mass index; BW: body weight; BOT: basal oral therapy; OAD: oral antidiabetic agents; SU: sulfonylurea; BG: biguanide; TZD: thiazolidine; αGI: α-glucosidase inhibitor.

This study was an observational, retrospective study that aimed to evaluate the efficacy and adverse events associated with lira after switching from insulin therapy.

### Statistical analysis

Data are presented as means ± SD. One-way ANOVA and the Bonferroni post hoc test were used for comparisons of the numerical values of HbA1c, FPG, PPG, and BW between baseline and after treatment. Statistical analyses included the paired t-test, one-way ANOVA, the χ^2^ test for categorical variables, and univariate linear correlation. Multiple linear regression analysis was used to identify variables that were independently associated with differences in HbA1c and BW reduction at 12 and 24 weeks. To identify clinical factors predictive of the efficacy of switching from insulin to lira, logistic regression analyses were conducted for age at baseline, sex, duration of diabetes, BW, baseline HbA1c, fasting and postprandial plasma glucose, SBP, and diastolic blood pressure (DBP). All data were analyzed using SPSS statistical software version 18 for Windows (SPSS Japan Inc., Tokyo, Japan). Significance was defined as a two-tailed P ≤ 0.05.

## Results

### Clinical characteristics in the continuation and discontinuation groups

The clinical characteristics in which a significant difference was seen between the continuation and discontinuation groups were: duration of diabetes (years) (continuation group: 11 ± 8, discontinuation group: 14 ± 9), baseline HbA1c (continuation group: 7.3 ± 1.2%, 56 ± 13.1 mmol/mol; discontinuation group: 8.0 ± 1.4%, 64 ± 15.3 mmol/mol), change in Hb1Ac from baseline to 12 weeks (ΔHbA1c 0 - 3 m) (continuation group: -0.4 ± 0.3%, -4.4 ± 3.3 mmol/mol, discontinuation group: 0.6 ± 0.9%, 6.6 ± 9.8 mmol/mol), and baseline daily insulin dose (U/day) (continuation group: 18 ± 15, discontinuation group: 29 ± 17). No significant differences were seen in age, BMI, blood pressure, lipids, number of patients using each oral antidiabetic agent before the start of lira, or number of patients using each type of insulin formulation (P > 0.05) (Table 1).

### Trends in blood glucose and BW in the continuation group

In the continuation group HbA1c (NGSP, IFCC) was significantly decreased at 12 weeks of lira administration (7.0 ± 1.3%, 53 ± 14.2 mmol/mol) compared with baseline (7.4 ± 1.25%, 57 ± 13.7 mmol/mol) (P < 0.0001).

On multiple regression analysis with ΔHbA1c as the dependent variable in the continuation group, there was a significant negative correlation with baseline HbA1c at 12 weeks of lira administration (P < 0.01), and a significant positive correlation with baseline daily insulin dose (P < 0.001). On multiple regression analysis with ΔBW as the dependent variable in the same group, a significant negative correlation was seen with baseline daily insulin dose (P < 0.001) (Table 2).

**Table 2:** Predictors of ΔHbA1c and ΔBW in the Liraglutide Therapy Groups on Multiple Regression Analysis

	Predictors of ΔHbA1c 0 - 12 weeks		Predictors of ΔBW 0 - 12 weeks	
	β	P	β	P
Age	-0.034	0.665	-0.102	0.190
Sex	-0.062	0.396	0.061	0.425
Duration	-0.015	0.839	-0.024	0.749
BMI	0.032	0.704	-0.052	0.539
Basal HbA1c	-0.201	< 0.01	-0.084	0.339
Total insulin dose	0.018	< 0.001	-0.068	< 0.001

BMI, body mass index.

### Number of days of lira administration and reasons for discontinuation in the discontinuation group

The mean number of days of lira administration in the discontinuation group was 68 days (maximum 160 days, minimum 2 days). The number of days of lira administration was significantly shorter in the group with gastrointestinal complications (42.4 ± 41.1 days) than in the group without gastrointestinal complications (81.0 ± 49.8 days). At the time of discontinuation, HbA1c was 8.3 ± 2.0%, 67 ± 21.9 mmol/mol, significantly worse than the baseline HbA1c. The reason for discontinuation was poor blood glucose control in 33 patients (47.1%), adverse events in 27 patients (38.6%) (loss of appetite in 10 patients, nausea and vomiting in 7 patients, myocardial infarction in 2 patients, epigastric pain, heartburn, hepatic dysfunction, cerebral infarction, and pneumonia in 1 patient each, and unknown in 3 patients), improved blood glucose control in 1 patient (1.4%), and other reasons in 9 patients (12.9%). No patients developed diabetic ketoacidosis or severe hypoglycemia during therapy.

### Factors affecting the advisability of continuing lira

**Figure 1:**
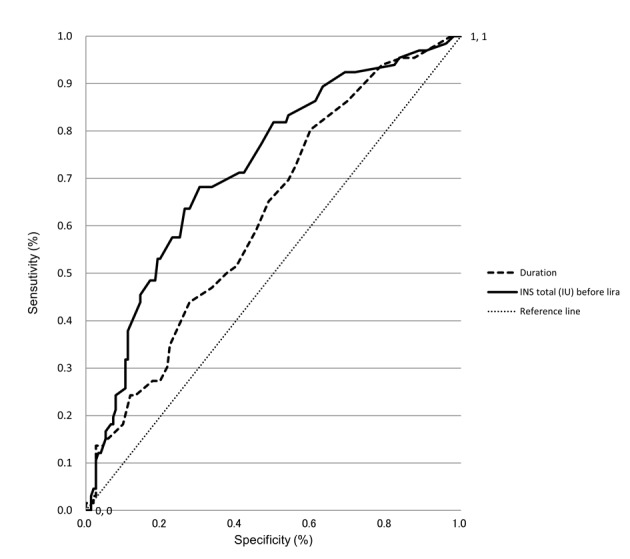
ROC curve analyses to identify predictors of continuing lira therapy. ROC curves of duration and total insulin dose before start of liraglutide therapy as covariates in the prediction of 6-month continuation of lira therapy are shown. The higher the area under the curve (namely, the greater the curvature away from the 50% line (zero prediction)), the greater the predictive power.

Factors affecting the advisability of continuing lira were investigated using a logistic regression analysis with advisability of continuing lira as a dependent variable. Significant relationships were seen with baseline daily insulin doses (P < 0.001) and duration of diabetes (P < 0.05) (Table 3). Based on the receiver operating characteristic (ROC) curve analyses, cut-off values of 19 units for daily insulin dose and nine years for duration of disease were identified (Fig. 1).

**Table 3:** Logistic Regression Analysis With Advisability of Continuing Liraglutide Therapy to 6 Months as the Dependent Variable

	OR	95% CI	P value
Duration	1.04	1.005 to 1.076	0.024
Total insulin dose	1.04	1.016 to 1.056	< 0.001

## Discussion

Problems in treatment using sulfonylurea drugs and other existing oral antidiabetic agents or insulin typically include weight gain and hypoglycemia, but when incretin formulations are administered alone, the risk of weight gain and hypoglycemia is known to be low [8, 9]. GLP-1 receptor agonists, like insulin, are injectable formulations. However, it is reported that, with GLP-1 receptor agonists, the timing of injections can be established freely without relation to meals, the dosage is constant, patient satisfaction is high, and there is a weight reduction effect. On the other hand, a switch from insulin to GLP-1 receptor agonists is difficult in many cases when the duration of disease is long and the insulin dose is high.

In fact, the discontinuation group in the present study had a longer duration of diabetes (mean 14 years), higher baseline HbA1c (mean 8%, 64 mmol/mol), and higher baseline insulin dose (mean 29 U/day) than the continuation group. It is known that the GLP-1 receptor expression level in the islets of Langerhans decreases in patients who have had elevated blood glucose for many years [10], and we conjectured that improvement or maintenance of blood glucose control was not achieved with lira and led to discontinuation in the discontinuation group as a result of a similar phenomenon. The mean number of days of lira administration was 68 days in the discontinuation group, but gastrointestinal complications were seen to cause a significant decrease in the number of days that it could be administered. Even among the adverse events that accounted for about 39% of the reasons for discontinuation, gastrointestinal symptoms such as loss of appetite were seen in 66.7%. Sufficient attention to the appearance of gastrointestinal symptoms and measures such as appropriate dosage adjustments are therefore necessary.

In the continuation group, significant improvement in HbA1c, as well as significant weight loss, was seen during therapy. The results of multiple regression analysis with ΔHbA1c as the dependent variable in the continuation group indicated that HbA1c at 12 weeks of lira administration decreased with higher baseline HbA1c and increased with higher baseline daily insulin doses. The association of total insulin dosages with liraglutide efficacy can be inferred based on the fact that decreased β-cell function requires increased dosages of total insulin. Therefore, it is reasonable to assume that liraglutide efficacy is clinically predicted by total insulin dosage. The results of multiple regression analysis with ΔBW as the dependent variable indicated that ΔBW at 24 weeks of lira administration was higher with higher baseline daily insulin doses and longer duration of diabetes.

Currently, there is no fixed view on factors that affect the advisability of lira continuation after switching from insulin therapy to lira. However, factors that have been reported to predict the efficacy of lira in type 2 DM patients include diabetes duration of 19.5 years, C-peptide index (CPI) of 1.1, insulinogenic index (II) of 0.14, fasting C-peptide (F-CPR) of 1.5 ng/mL, and urine C-peptide (U-CPR) of 33.3 μg/day [11]. Insulin dose and blood glucose level after breakfast are also useful predictors of lira efficacy in routine practice, and it has been suggested that they reflect the remaining function of pancreatic β cells [12].

We therefore conducted an investigation of the cut-offs for baseline daily insulin dose and duration of disease (P < 0.05) using the area under the ROC curve, and they were found to be a daily insulin dose of 19 units and disease duration of nine years. These findings may be predictors of the advisability of switching from insulin therapy to lira and continuing that treatment in Japanese type 2 DM patients.

As in previous clinical trials [4, 7, 13] gastrointestinal side-effects were observed in the present study. However, no associations between the occurrence of gastrointestinal side-effects and any baseline characteristics were observed (data not shown). Therefore, it might be difficult to predict the likelihood of adverse events. No major adverse events such as ketoacidosis were seen during the course of the present study, but there have been cases in Japan of diabetic ketoacidosis or hyperglycemia following a switch from insulin therapy to lira. Thus, the published safety information and revised usage warnings for lira by the Ministry of Health, Labor and Welfare are still fresh in our memory. Careful attention to the patient’s clinical condition is needed when switching from insulin therapy to lira.

The major limitations of the present study are its retrospective design and the limited number of patients studied. In addition, almost all of the patients switched therapy from other OHAs to lira; therefore, a suitable control group was not included. Other markers, such as waist circumference as a marker of central obesity, markers for assessment of β-cell function, and basal active GLP-1 levels, that could have contributed to the HbA1c reduction with lira were not measured in the present study.

In conclusion, the change from insulin to lira therapy resulted in favorable outcomes for some patients who were insufficiently controlled with the usual insulin therapy. It is thought that proper and safe treatment can be expected by carefully selecting patients based on duration of disease and insulin dose when switching from insulin therapy to lira in type 2 DM patients, which will expand treatment options. However, the efficacy and tolerability of the change from insulin to liraglutide therapy must be validated in future prospective studies.
